# Clinical Profile and Outcome of Dengue Fever in Medical Intensive Care Unit of a Tertiary Level Hospital: An Observational Study

**DOI:** 10.31729/jnma.9080

**Published:** 2025-06-30

**Authors:** Eliza Koirala, Gopendra Prasad Deo, Shital Adhikari, Basanta Gauli, Akriti Bajracharya

**Affiliations:** 1 Department of Critical Care Medicine, Chitwan Medical College Teaching Hospital, Bharatpur, Chitwan, Nepal; 2 Department of Anesthesiology and Critical Care Medicine, Chitwan Medical College Teaching Hospital, Bharatpur, Chitwan, Nepal; 3 Department of Pulmonology and Critical Care Medicine, Chitwan Medical College Teaching Hospital, Bharatpur, Chitwan, Nepal

**Keywords:** *clinical profile and outcome*, *dengue fever*, *medical intensive care unit*, *multi-organ disorder*, *Nepal*

## Abstract

**Introduction:**

Dengue fever poses a significant public health challenge in Nepal, with increasing severe cases requiring intensive care. Limited data exist on the clinical profile and outcomes of critically ill dengue patients in resource-limited settings. This study is aimed to characterize the clinical features, outcomes and complications of dengue patients admitted to a tertiary Intensive care unit in Nepal.

**Methods:**

A prospective observational study was conducted at from August to September 2024 after ethical approval. We enrolled 104 serologically confirmed dengue patients (aged ≥16 years) admitted to the Medical Intensive care unit. Exclusion criteria included co-infections with other tropical diseases. Data on demographics, clinical presentation, laboratory parameters (including platelet counts, liver enzymes), organ dysfunction (SOFA score), and outcomes were collected. Descriptive data analysis was used to report frequency, proportion, measure of central tendency and measure of dispersion based on nature of data.

**Results:**

The cohort (median age 41 years, 54.8% male) exhibited severe manifestations: thrombocytopenia (median platelets count 49,000/μL; IQR: 30,250-85,000 per μL), hepatic injury 86 (82.7%); median AST 176.5 U/L, ALT 208.5 U/L), and moderate organ dysfunction (median SOFA score 4). Hypertension 27 (25.96%) and diabetes 23 (22.12%) were common comorbidities. Despite 22 (21.2%) developing multi-organ dysfunction syndrome, mortality was remarkably low 1 (0.96%).

**Conclusions:**

Dengue fever cases peak during the month of August and September in endemic areas (Chitwan and surrounding district). Mortality could be reduced in severe dengue with appropriate critical care, highlighting the need to strengthen ICU capacity in endemic regions.

## INTRODUCTION

Globally, dengue affects over 7.6 million people annually, with critical cases leading more than 3000 deaths.^1^ There are clear seasonal patterns in dengue incidence in South-East Asia, with Nepal reporting increasing monsoon-driven cases.^1,2^ Despite this burden, care remains largely supportive and expert opinion-based, with few therapeutic advances. Severe dengue frequently requires Intensive Care Unit (ICU) admission due to multi-organ failure, presenting with fever, thrombocytopenia, bleeding, and pain symptoms.^3^

The variability in clinical progression and intensive care needs highlights management challenges. There is limited data on clinical profile, complications, and outcomes of severe dengue cases in ICU settings^3,4^ This study is crucial for improving patient outcomes and reducing mortality rates associated with severe dengue in Nepal.

The aim of the study was to explore the clinical profile and outcome of the dengue positive cases admitted in ICU of a tertiary level hospital.

## METHODS

This was a hospital-based observational study conducted among dengue patients admitted at the Medical Intensive Care Unit (MICU) of Chitwan Medical College, Teaching Hospital, Baharatpur,Nepal. The MICU consists of two units MICU I and step down unit MICU II. There are a total of 30 beds, serving as a referral center for critically ill patients in central Nepal. Ethical approval (Reference number: CMC IRC/081/082/104) was obtained from CMCTH with written consent from patients or applied consent from legally authorized representatives for patient who were not able to provide consent and confidentiality was maintained through anonymized data collection.

All patients- who were dengue serology positive, aged ≥16 years and were admitted to MICU from 1st August to 30th September 2024 were included. Co-infected patients with other tropical fevers like scrub typhus, influenza, malaria, leptospirosis, and typhoid fever were excluded. Non-structural (NS1) antigen strip® ELISA (BioRad), Immunoglobulin (IgM) ELISA were used for dengue serology testing. Data were collected prospectively from medical records of patients diagnosed with serologically proven dengue fever, using standardized structured proforma. Baseline characteristics included demographics (age and sex), comorbidities (hypertension, diabetes mellitus, coronary artery disease and others), and clinical presentation (fever; gastrointestinal symptoms-abdominal pain, loss of appetite, vomiting and loose stool; neurological symptoms- headache, altered sensorium; hematological symptoms- bleeding, jaundice; and others symptoms- myalgia, shortness of breath, syncope). The Sequential Organ Failure Assessment (SOFA) score was calculated at the first 24 hours of ICU admission.^5^

While the SOFA score is not a dengue-specific classification, it was employed to quantify the degree of multi-organ dysfunction, which correlates with disease severity in critically ill dengue patients. Intensive care unit -specific parameters like SOFA scores and laboratory markers (e.g., platelet counts, liver enzymes) were used to characterize severity. Charts from ICU and laboratory reports were used to collect information that includes hemoglobin level, White Blood Cell Count (WBC), platelets count, International Normalised Ratio (INR), bilirubin level, transaminases levels - aspartate transaminase (AST) and alanine transaminase (ALT), and serum creatinine level. Patient management was according to WHO , Surviving Sepsis Campaign Guidelines 2021 and established critical care standards practices in MICU.^. 6^ The outcome was assessed through clinical outcome (ICU in-hospital mortality , Left Against Medical Advice - LAMA, Referral and discharge), and complication (hepatic injury, pleural effusion, Multiple Organ Dysfunction Syndrome- MODS, Acute Kidney Injury- AKI, Dengue shock syndrome- DSS, Acute respiratory distress syndrome- ARDS, encephalopathy, myocarditis and coagulopathy). In hospital mortality was considered upon the death of the patient while in the hospital. Patients were considered LAMA when the patient or their attendent decided to take the patient before completion of the treatment of without patient being ready for discharge. Similarly referral was considered when patients were referred from the institution to other institution. Operational definitions followed World Health Organization (WHO) and critical care standards (e.g., thrombocytopenia as platelets <100×10^3^/L, ARDS by Berlin criteria). Methodological rigor was ensured through prospective data collection by researchers themselves; standardized laboratory testing (BioRad ELISA kits); daily quality checks; and adherence to STROBE guidelines.^7^

Data was analysed by using IBM SPSS Statistics for Windows, version 26 (IBM Corp., Armonk, N.Y., USA). Descriptive statistics such as frequency, percentage, measure of central tendency and measure of dispersion based on data nature, eg mean±standard deviation (SD) for normally distributed data or median (inter quartile range (IQR) for skewed data was used.

## RESULTS

A total of 104 patients with a diagnosis of dengue fever were admitted at MICU during the studied period (from 1^st^ August to 30^th^ September 2024). Patient swere from Chitwan 54 (51.92%), Nawalpur 9 (8.65%), Tanahun 26 (25.00%), Gorkha 9 (8.65%), Makwanpur 3 (2.89%), Parsa 1 (0.96%) and other districts 2 (1.92%). A median age of dengue patients was 41 years (IQR: 31.25-54.75 years) with 57 (54.80%) male.

Hypertension was present in 27 (25.96%) patients and diabetes in 23 (22.12%) patients. Fifteen (11.70%) patients had both hypertention and diabetes and 18 (14.10%) patients had multiple comorbidities. Clinically, 103 (99.00%) patients presented with fever, 49 (47.10%) had headache and 10 (9.60%) had bleeding manifestation. The median SOFA score was 4 (IQR: 3-5), and NS1 antigen positivity was detected in 98 (94.2%) cases. The median duration of fever was 4 (IQR: 3-5) days. There were 5 cases with scrub typhus coinfection and were excluded. (Table 1).

**Table 1:** Clinical feature of dengue patients admitted at the MICU in CMCTH (n=104).

Variables	n(%)
Comorbidity^*^	
Hypertension	27(25.96)
Diabetes Mellitus	23(22.12)
Ischaemic Heart Disease	4(3.85)
Others	13(12.50)
Fever	103(99.00)
GI symptoms^*^	
Abdominal pain	45(43.30)
Loss of appetite	64(61.50)
Vomiting	55(52.90
Loose stool	33(31.70)
Neurological symptoms^*^	
Headache	49(47.10)
Altered sensorium	4(3.80)
Hematological symptoms^*^	
Bleeding	10(9.60)
Jaundice	2(1.90)
Other symptoms^*^	
Myalgia	62(59.60)
Shortness ob breath	18(17.30)
Syncope	16(15.40)
**SOFA score** Serology pattern	
NS1	98(94.23)
IgG	1(0.96)
IgM	5(4.81)

* Multiple-response question; SOFA=Sequential Organ Failure Assessment; MICU=Medical Intensive Care Unit, NS1= Non Structural Antigen ; IgG=Immunoglobulin G; IgM=Immunoglogulin M; CMCTH=Chitwan Medical College Teaching Hospital

**Table 2:** Laboratory parameters of dengue cases admitted at the MICU in CMCTH (n=104).

Variables	Median (IQR)
Hemoglobin in g/dL	12.25(14-11)
WBC count per μL	3045(4125-2265)
Platelets counts per μL	49000(85000-30250)
INR	1(1.10-0.90)
Total bilirubin in mg/dL	0.6(0.80-0.42)
Creatinine in mg/dL	1 (1.2-0.8)
ALT in U/L	208.5(629.75-104.75)
AST in U/L	176.5(412.25-73)

WBC= White Blood Cell; ALT=Alannine Aminotransferase; AST=Aspartate Aminotransferase; INR= International Normalized Ratio; CMCTH=Chitwan Medical College Teaching Hospital

Laboratory findings revealed thrombocytopenia with median platelets count 49,000/μL (IQR: 30,250-85,000 per μA) and median values for liver enzymes were AST; 176.5 U/L (IQR: 73-412.25 U/L); ALT 208.5 U/L, (IQR: 104.75-629.75 U/L) (Table 2).

Hepatic injury 86 (82.70%), pleural effusion 40 (38.50) and MODS 22 (21.20%) were the most frequent complications (Table 3). Mortality was 1 (0.96%), with 91 (87.50%) patients discharged (Table 3).

**Table 3:** Outcomes of dengue cases admitted at the MICU in CMCTH (n=104).

Variables	n(%)
Complications^*^	8 (82.70)
Hepatic injury	40 (38.50)
Pleural effusion	22 (21.20)
Multi organ dysfunction syndrome	20 (19.20)
Acute kidney injury	20 (19.20)
Dengue shock syndrome	9 (8.70)
Acute respiratory distress syndrome	5 (4.80)
Encephalopathy	4 (3.80)
Myocarditis	3 (2.90)
Coagulopathy	91 (87.50)
**Clinical outcome**	
Death	1 (0.96)
Discharge	12 (11.54)
Left against medical advice/Referral	

* Multiple-response question

## DISCUSSION

This study provides a comprehensive analysis of 104 patients with severe dengue requiring intensive care at a tertiary hospital in Nepal, which lies in dengue endemic area. Our findings reveal clinically significant patterns that contribute to the understanding of dengue's critical care burden in endemic regions. The cohort, predominantly middle-aged males, exhibited characteristic features of severe dengue, including thrombocytopenia, hepatic injury, and moderate organ dysfunction at admission.^8,9^ Notably, despite these severe manifestations, mortality remained remarkably low (<1.0%), suggesting the potential benefits of maintaining standard protocol for ICU care in resource-limited settings.^10,11^

Our study shows the median age of dengue patients was 41 years (IQR: 31.25-54.75 years) with 57 (54.80%) male. This is consistent with the findings from the studies from Mizoram, India, indicate that the majority of dengue patients are adult males, with a mean age of 42.08 years.^12^ Similarly, in Bangladesh, over 80% of dengue patients were male, with a significant proportion aged between 30 and 59 years.^13^ The demographic profile aligns with established patterns of dengue transmission in South Asia, where adult males face higher exposure risks due to occupational and environmental factors.^14^ The median age of 41 years reflects the working age population most vulnerable to severe dengue complications in Nepal. The male predominance (54.8%) may also reflect gender disparities in healthcare access or biological factors influencing disease severity.^15^

Laboratory findings offer important pathophysiological insights. The profound thrombocytopenia (median 49,000/μA) and elevated transaminases (AST 176.5 U/L, ALT 208.5 U/L) demonstrate the multisystem nature of severe dengue.^16-18^ The lowest platelet count was as low as 5000/μL and the highest level of ALT was as high as 13,000U/L. These values exceed those reported in many Southeast Asian studies, possibly indicating unique characteristics of circulating dengue strains in Nepal or differences in host immune responses.^19^ This is evidenced by the emergence of multiple serotypes and genotypes, as well as significant differences in host immune responses ob-served in the region.^2,20^

The high prevalence of hepatic injury (82.7%) particularly warrants attention, a it suggests po-tential viral tropism for liver tissue or immune mediated damage. The high prevalence of hepatic injury in dengue cases, particularly in ICU settings, suggests a significant interaction between the dengue virus and liver tissue, potentially due to viral tropism or immune mediated damage. Hepatic involvement in dengue is a well-documented phenomenon, with liver dysfunction ranging from mild transaminase elevation to severe hepatocyte injury. This liver damage is more pronounced in severe cases of dengue, such as dengue hemorrhagic fever, indicating a correla-tion between disease severity and liver involvement.^16,21^ This indicates that monitoring liver function is crucial in managing dengue patients, as liver dysfunction is a common complication. Elevated transaminases, hypoalbuminemia, and altered A:G ratios are useful biochemical markers for detecting and monitoring hepatic dysfunction. It is supported by elevated liver enzymes (median AST 176.5 U/L, IQR: 73-412.25 U/L; ALT 208.5 U/L, IQR: 104.75-629.75 U/L) reported by this study finding. Elevated levels of AST and ALT often signify hepatocellular damage, commonly seen in conditions like viral hepatitis and drug induced liver injury.^22^ The severity of liver dysfunction is evaluated through tests like total bilirubin, albumin, and international normalized ratio, which reflect liver health and function.

The clinical outcomes present an interesting paradox. While dengue patients frequently develop significant organ dysfunction (median SOFA score in this study), mortality rates in Nepal (0.96%) and parts of India remain notably lower than other Asian regions.^23^ This might reflect early ICU interventions (fluid restriction not resuscitation resuscitation, platelet monitoring), standardized protocols, and possible differences in circulating strains (dominance of DENV-2 with regional variations in virulence). Use of point of care ultrasound to assess capillary leak and guide early vasopressor and restricted fluid management is the mainstay in management. Few cases with prolonged fever beyond 7 days received dexamethasone practised as individualized patient management based on case reports and case series, during patient management, N acetylcysteine (NAC) was given to patients with liver enzymes >1000U. For patients who had significant nausea, vomiting and anorexia, lower cutoff od 800U was used for NAC infusion. However, the lack of long-term follow-up, particularly for LAMA cases, may underestimate true mortality. In our study, LAMA cases were those who improved clinically but still had either low platelet or elevated liver enzymes. Strengthened ICU capacity and adherence to WHO guidelines likely to identify severe dengue and dengue with warning signs for early ICU admis-sion contribute to these improved outcomes in Nepal.

There were some interesting and atypical cases, one patient had acute liver failure who was managed with two sessions of plasmapheresis, he improved and was discharged without com-plication, the other male patient presented with sudden collapse was intubated and had longest ICU stay. His MRI brain showed cerebellitis, he was also discharged without residual organ disfunction. One young boy had to be operated and surgically managed for gross hemoperitoneum that mimicked ST elevation MI. However, this study does not discuss outcome according to severity of cases, which limit the complete contextual understanding of outcome.

While the study's strengths include its prospective design and comprehensive assessment of dengue's critical care burden in Nepal's tertiary hospital system, limitations such as single-center design, short study duration, and lack of long-term follow-up for outcome. Without longterm follow-up, the true mortality rate might be underestimated (e.g., if some LAMA patients died after leaving). Future research should explore multicenter studies across Nepal's diverse ecological zones, considering follow-up for LAMA cases. It would have been better if the clinical outcome eg mortality could be discussed as per severity of dengue itself.

## CONCLUSIONS

Most of the patient in this study were middle-aged males, with high hepatic involvement and low mortality rates.
